# Implantable theranostic device for in vivo real-time NMR evaluation of drug impact in brain tumors

**DOI:** 10.1038/s41598-024-55269-1

**Published:** 2024-02-24

**Authors:** Justine Deborne, Imad Benkhaled, Véronique Bouchaud, Noël Pinaud, Yannick Crémillieux

**Affiliations:** 1 Institut des Sciences Moléculaires, Université de Bordeaux, UMR 5255, Bordeaux, France; 2 Centre de Résonance Magnétique des Systèmes Biologiques, Université de Bordeaux, UMR 5536, Bordeaux, France

**Keywords:** Magnetic resonance imaging, Cancer imaging

## Abstract

The evaluation of the efficacy of a drug is a fundamental step in the development of new treatments or in personalized therapeutic strategies and patient management. Ideally, this evaluation should be rapid, possibly in real time, easy to perform and reliable. In addition, it should be associated with as few adverse effects as possible for the patient. In this study, we present a device designed to meet these goals for assessing therapeutic response. This theranostic device is based on the use of magnetic resonance imaging and spectroscopy for the diagnostic aspect and on the application of the convection-enhanced delivery technique for the therapeutic aspect. The miniaturized device is implantable and can be used in vivo in a target tissue. In this study, the device was applied to rodent glioma models with local administration of choline kinase inhibitor and acquisition of magnetic resonance images and spectra at 7 Tesla. The variations in the concentration of key metabolites measured by the device during the administration of the molecules demonstrate the relevance of the approach and the potential of the device.

## Introduction

Chemotherapeutic or immunotherapeutic agents are mainly administered to cancer patients via systemic routes (intravenous injection, oral absorption…). These systemic administrations, which result in the distribution of the anticancer drug throughout the body, are faced with certain difficulties and adverse side effects that sometimes limit their efficacy and applicability. In order to achieve a cytotoxic intratumoral concentration, the anticancer drugs are used at the maximum tolerated dose and their toxicity on vital organs (kidney, heart, central nervous system, …) and cells (hematocytes, skin, …) can be problematic in debilitated patients. In the case of brain tumors, the efficacy of systemically administered therapies may be further reduced by the blood–brain barrier (BBB), which prevents the passage of some drugs from the bloodstream into the extracellular fluid of the central nervous system.

To overcome the limitations and drawbacks of systemic administration of anticancer drugs, intratumoral chemotherapy and immunotherapy are alternative approaches that are being actively pursued and investigated in preclinical research and clinical trials for clinical applications^1–3^. The potential benefits of intratumoral administration are numerous. It avoids the passage of the drug through the blood compartment and the associated inconveniences such as dilution, binding to blood proteins, hepatic first-pass effect, blood–brain and vascular-tumor barriers. It allows the use of lower doses and therefore has a limited general toxicity.

Although intratumoral administration of anticancer drugs is a promising approach, it is an invasive procedure that can present some difficulties with respect to the injection process (flow rate, dose, access to the tumor), the variability of the drug dispersion and pharmacokinetics in a heterogeneous tumor, and leakage into surrounding tissues^4,5^.

Convection-enhanced delivery (CED) techniques, which involve continuous drug delivery through infusion catheters and the application of a hydrostatic pressure gradient, are commonly used to facilitate drug distribution via convective flow within the solid tumor^6–8^.

When a drug delivery catheter is placed in a tumor, it seems desirable to associate it with a local analysis or imaging device capable of performing a number of useful measurements to assess the efficacy of the treatment and, possibly, improve its success. This associated diagnostic tool should ideally be used to assess the biodistribution of the drug within the tumor and to monitor and quantify the impact of the therapeutic drug on the tumor cells and their microenvironment. In addition, this imaging device could be used to ensure proper positioning of the delivery device.

Among the in vivo imaging techniques, magnetic resonance imaging (MRI) and the associated magnetic resonance spectroscopy (MRS) techniques are two of the most appropriate candidates to achieve these goals. These two essential NMR-based techniques are widely used in oncology and pharmacology because of their ability to provide critical information about the tumor microenvironment and tumor metabolism. MRI offers a wide range of soft tissue contrast and quantitative measurements (relaxometry, diffusion, oxygenation, pH, temperature,…) while MRS has the ability to detect differences in metabolite concentrations between healthy tissue and tumor. Together, reliable MRI and MRS biomarkers allow to study the therapeutic response and thus to monitor and evaluate the antitumor efficacy of an administered drug^9,10^.

However, standard MRI and MRS protocols are characterized by a relatively low detection sensitivity, resulting in a typical millimer spatial resolution that is not suitable for a precise and fine assessment of the therapeutic effect of molecules delivered locally by CED. One solution to this sensitivity limitation is to design a dedicated NMR coil whose dimensions correspond to the volume to be examined, and to position it as close as possible to the region of interest. Reducing the size of the RF coil advantageously translates into an increase in coil sensitivity per unit volume, while positioning it in close proximity to the region of interest optimizes detection sensitivity in this region^11–15^.

The approach used in this study was to integrate the NMR detection coil and the capillary for CED into a single implantable device, combining drug delivery and NMR diagnostic capabilities.

Here, we report the in vitro and in vivo performance of this theranostic device and present the results of its evaluation and application in a rodent animal model of glioblastoma with CED of choline kinase inhibitor coupled with the acquisition of in vivo MR images and MRS proton spectra.

## Results

### Fabrication, integration and implantation of the device

**Figure 1:**
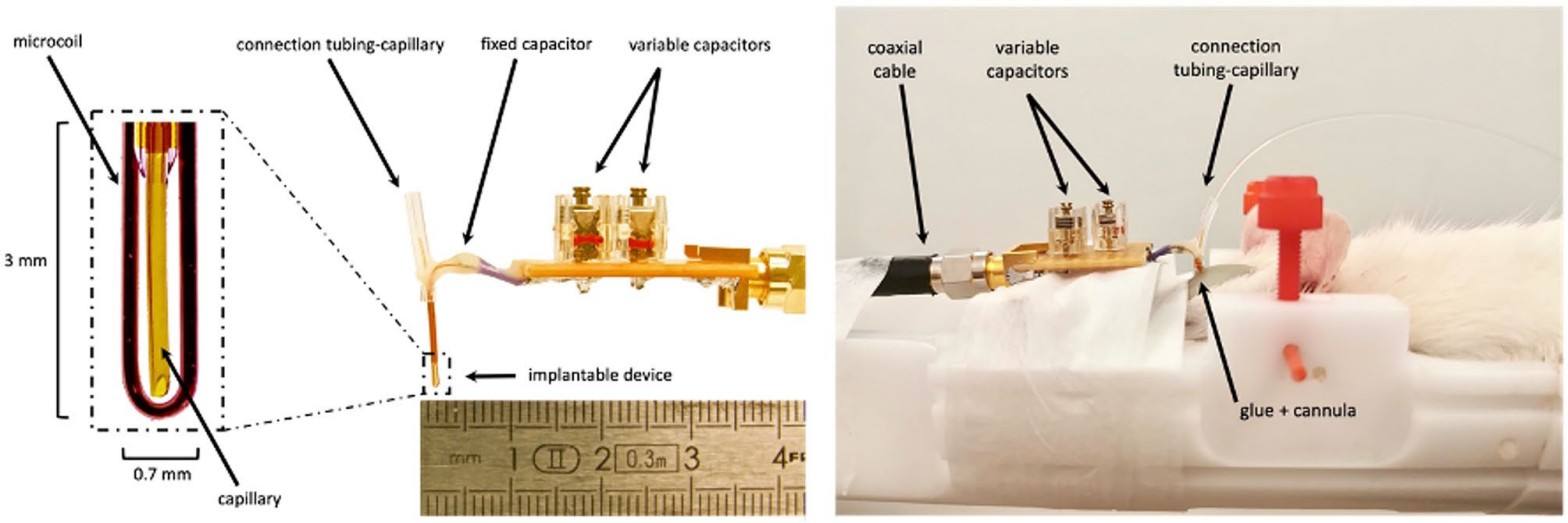
Overview of the implantable device and in vivo experimental set-up. On the left, the theranostic device composed of the capillary for CED and the microcoil with its circuit board and capacitors. The zoomed part in the rectangle corresponds to the part implanted in tissue. On the right, an overview of the experimental set-up with the animal positioned in the MRI cradle with the implanted device in the brain.

An overview of the implantable device including an NMR microcoil and a capillary for intratumoral CED of drug is shown in Fig. 1. To realize the NMR detection microcoil, the copper wire was bent to form an elliptical-shape loop with typical outer dimensions of 3 mm for the long axis, 700 µm for the short axis and 150 µm thickness. The capillary used for the CED was positioned over a length of 2.7 mm in the center of the loop formed by the wire. Both ends of the copper wire and the capillary were then passed through a 5-mm long polyimide tubing which was afterwards sealed. The endings of the wire were soldered to a circuit board with fixed and variable capacitors approximately 1 cm away from the tubing ending.

In order to implant the device, a cannula was fixed to the animal’s skull above the region to be investigated. One day after the cannula was placed, the device was inserted into the cannula and implanted into the brain with the animal lying in prone position in the MRI animal bed. The circuit board was then connected to the preamplifier of the spectrometer via a 2-m coaxial cable. The animal bed was then moved inside the magnet with the microcoil positioned at the magnetic isocenter. The variable capacitors on the circuit board were used for matching at 50 Ω and for tuning of the microcoil at the proton Larmor frequency of 300 MHz corresponding to the static magnetic field of 7 T. The capillary inlet was connected to a 1.5-m-long flexible tubing, which in turn was connected to a syringe pump for drug injection. An overview of the experimental set-up with the animal positioned in the MRI cradle is shown in Fig. 1.

### Assessment of microcoil performance

The spatial distribution and the amplitude of the B_1_ RF magnetic field generated by the microcoil were simulated using an electromagnetic simulation software based on the finite element method (FEM). The elliptical shape of the microcoil generated an RF magnetic field close to that generated by surface coils. 3D displays of the orientation and amplitude of the simulated B_1_ magnetic field obtained with the FEM modeling software are shown in Fig. S1 in supplementary information. The isosurfaces of the amplitude of the transverse component, relative to the static magnetic field, of the B_1_ RF field are shown in Fig. S2 in supplementary information. An empirical and arbitrary threshold for the detection sensitivity of the microcoil was set at 30% of the maximum amplitude value of this transverse B_1_ RF field and was used to estimate the detection volume of the microcoil. Based on this definition, the detection volume of the microcoil was measured to be 1 $\upmu$L.

MRI was used to measure the detection volume of the manufactured microcoils. 3D isotropic ZTE MRI acquisitions were performed with the microcoil immersed in saline solution. Figure S3 in supplementary information shows the volume rendering of the detection volume of the microcoil. The shape of the detection volume was found to match with the one derived from FEM simulations shown in Fig. S2. To quantify the detection volume, the MRI signal intensity was thresholded at a value corresponding to 30% of the maximum signal intensity. The resulting volume was segmented and its value was found to be 1.1 $\upmu$L, in agreement with the volume obtained from the FEM simulation. The unloaded and loaded in vivo quality factors of the microcoils were measured to be equal to 55 and 50, respectively.

The detection sensitivity of the microcoil was compared with that of a commercial surface coil used for rat brain imaging. To this end, NMR proton spectra were acquired either with a 2 × 2 rat brain surface array coil or with the microcoil. Both coils were used as receiver only. A commercially available quadrature birdcage volume coil was used as a transmitter in both configurations. The acquisitions were performed on a saline solution with the addition, at a concentration of 25 mM, of metabolites present in the brain including choline, creatine, lactate and *N*-acetylaspartate (NAA). For these measurements, the surface coil was positioned over the metabolite solution at a distance equivalent to that encountered for an MRS acquisition in the rat brain, while the microcoil was immersed in the solution.

The dimensionless FOG (Factor of Gain) was used for comparing the detection sensitivity of the microcoil with that of the surface coil.

The FOG provided by the microcoil was computed as:

1 $$FOG{ } = { }\frac{{nLOD_{m} \left( {surface{ }coil} \right)}}{{nLOD_{m} { }\left( {microcoil} \right)}}$$

where the normalized mass limit of detection nLOD_m_ was defined as,

2 $$nLOD_{m} = \frac{{3 \cdot mol \cdot T_{acq}^{1/2} }}{SNR}$$

with mol, the number of moles of metabolites within the detection volume of the coil, T_acq_ the acquisition time and the SNR (signal-to-noise ratio) of the acquired spectra. The nLOD_m_, that can be expressed in $\upmu$mol s^1/2^, corresponds to the minimum number of mole, normalized by the acquisition time, that provides an SNR of 3 which is considered as a lower limit for NMR detection^16,17^. The values of FOG obtained for the doublet line of lactate (1.31 ppm relative to water peak) and the singlet lines of NAA (2.01 ppm), creatine (3.03 and 3.91 ppm) and choline (3.18 ppm) from the test solution are reported in Table 1. The FOG values ranged from 67 for creatine to 164 for NAA and the average FOG for the five resonances was equal to 119.

**Table 1:** FOG for the microcoil relative to a surface coil for rodent brain for five resonance lines of metabolites present in the brain.

Metabolites of interest	Lactate doublet 1.3 ppm	NAA 2 ppm	Choline 3.18 ppm	Creatine 3.02 ppm	Creatine 3.91 ppm
FOG (dimensionless)	74	164	154	134	67

The values correspond to the average value obtained from three sets of data.

### Evaluation of the CED of solution in vivo

The CED of molecules using the device’s capillary was investigated in vivo by administering an artificial cerebrospinal fluid (aCSF) solution containing Gd-based contrast agents. The device was inserted into the cannula and implanted in the brain of a healthy animal one day after the cannula placement.

**Figure 2:**
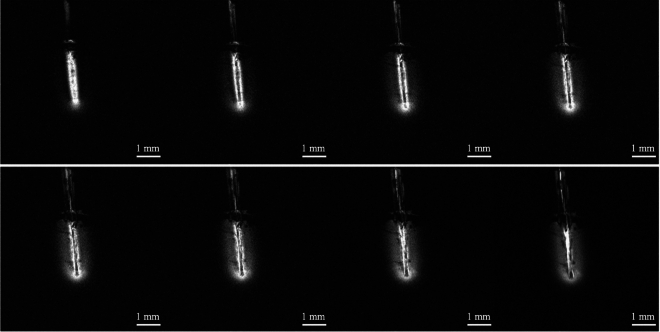
In vivo MRI of CED of contrast agent solution. Series of T1-weighted FLASH MRI images acquired with the implanted microcoil as a receiver. FLASH sequence was used with the following parameters: slice thickness = 0.4 mm, FOV = 10 × 10 mm^2^, in-plane resolution = 26 × 26 μm^2^, acquisition time = 8 min 19 s. The contrast agent solution was infused at a flow rate of 0.1 μL/min. The delay between each acquired image was 16 min. The first image, top row on the left was acquired before the start of the CED of the solution.

Before the implantation of the device in the rat brain, the capillary and its connecting tubing were filled with the contrast agent solution contained in the syringe used for infusion. Following device implantation, a continuous series of T_1_-weighted MRI images were acquired and upon completion of image acquisition at baseline, the contrast agent solution was infused at a flow rate of 0.1 µL/min. A selection of images acquired in the plane containing the capillary is shown in Fig. 2. Due to the detection sensitivity of the microcoil, tissues in the vicinity of the implanted device were visualized with voxel sizes of 0.27 nL and acquisition times on the order of 8 min, compatible with MRI studies on small animals. The use of a T_1_-weighted MRI sequence allowed visualization of the spatio-temporal evolution of the distribution of the T_1_-shortening contrast agent administered by CED within the brain tissue. The volume of detection of the microcoil was fully highlighted with the contrast agent solution approximately one hour after the start of the CED of the solution.

### Evaluation of the device in an animal model of brain tumor

In tumor-bearing animals, the cannula was fixed, two weeks after the tumor induction, to the animal’s skull above the brain tumor. The mean tumor volume was 94 ± 26 mm^3^ 16 days after stereotactic injection of tumor cells. In control and tumor animals the device was inserted in the cannula and implanted in the brain or in the tumor one day after the cannula placement.

**Figure 3:**
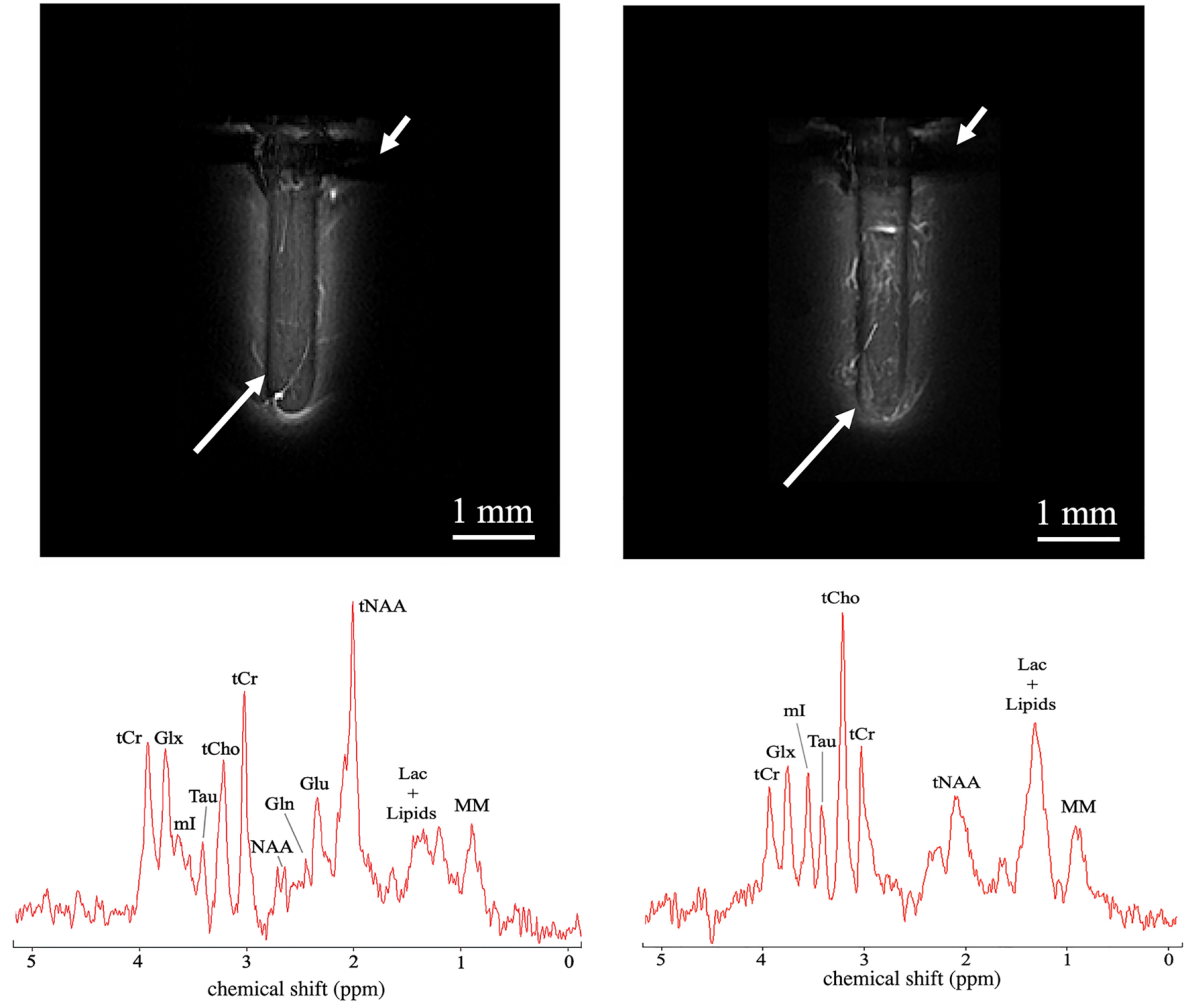
MRI and MRS in healthy brain tissue and glioblastoma. MIP images obtained in healthy brain tissue (left side) and C6 tumor (right side) using the microcoil as a receiver. In plane resolution of the MIP image was 33 μm. The short and long arrows are pointing hypointense regions corresponding to the skull and the coil wire respectively. Corresponding proton spectra obtained in healthy brain tissue (left side) and C6 tumor (right side) using the microcoil as a receiver and a PRESS sequence with a total acquisition time of 8 min and 32 s.

Figure 3 exemplifies the MR images and the MR proton spectra that were obtained in healthy brain tissue of control animals and in glioblastoma in tumor-bearing animals.

The MR images shown in Fig. 3 correspond to MIP (Maximal Intensity Projection) images obtained from a 2D multislice FLASH acquisition with five slices of 0.5 mm thickness, in plane resolution of 33 μm and a total acquisition time of 26 min 40 s. The diameters of the vessels observed in the MIP images ranged typically between 60 and 100 μm and were attributed to arterioles.

MR images obtained with the microcoil in healthy animals did not detect the presence of inflammation in the brain after implantation of the device.

Twelve NMR resonance lines, with Cramér-Rao lower bounds (CRLB) larger than 20% of the SNR, were identified in the corresponding proton spectra obtained in control and tumor-bearing animals acquired with the microcoil as a receiver. These included two resonances from creatine and phosphocreatine referred as total creatine (tCr), glutamate (Glu), glutamine (Gln) and composite glutamate-glutamine (Glx), myo-inositol (mI), lactate (Lac), *N*-acetylaspartate and *N*-acetylaspartylglutamate (tNAA), choline-containing compounds referred as total choline (tCho), taurine (Tau) and broad resonances corresponding to macromolecules (MM).

Notable differences were apparent between the two examples of spectra shown in Fig. 3 obtained in healthy brain tissue and tumor. In particular, there was a significant drop in the NAA peak, considered as a neuronal marker, and a large increase in the lactate/lipid and choline peaks, indicative of a cholinic phenotype of the tumor^20^.

To establish the differences in the metabolic profiles between healthy brain tissue and tumor, twelve control rats and eight C6 tumor-bearing animals in total were investigated with the implantable device. To eliminate inter-animal variations in MR signal intensity due to changes in experimental conditions, metabolite concentration ratios were compared between the two groups. The concentration ratios tCho/tCr, tNAA/tCr, tCho/tNAA, mI/tCr, Glx/tCr commonly used in neuro-oncology studies^21,22^ were compared.

**Figure 4:**
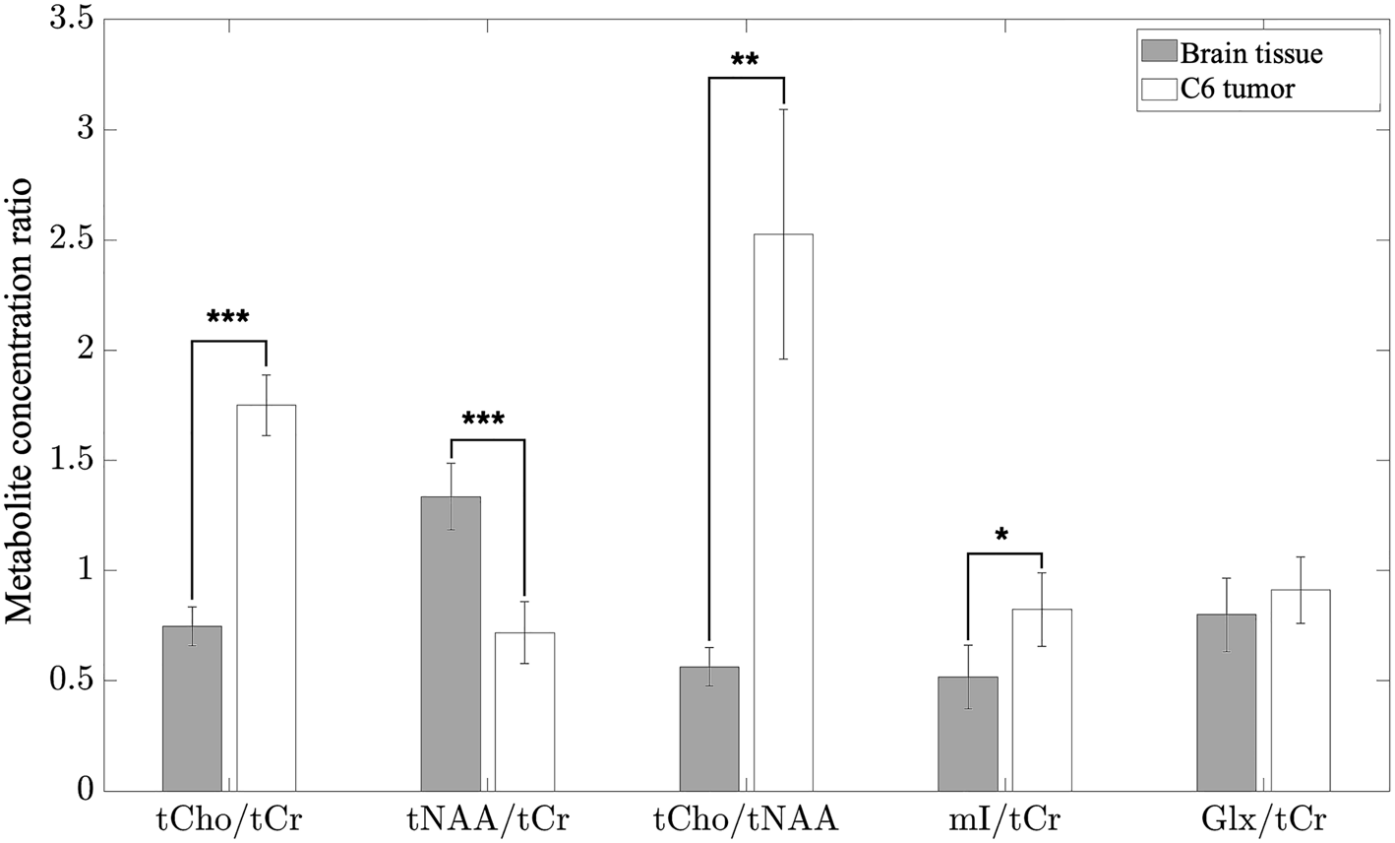
Quantification of metabolite concentration. Metabolite concentration obtained in the brain of 12 control animals and in C6 glioblastoma in 8 tumor-bearing animals. Non-paired t-test was applied for establishing statistical significance with * corresponding to p < 0.05, ** to p < 0.001 and *** to p < 0.0001. Error bars correspond to standard deviations of measured ratios in each animal group.

A non-paired t-test was applied to evaluate the differences in concentration ratios between the two groups. The results shown in Fig. 4 indicate statistical differences for all ratios to the exception of Glx/tCr. Very significant statistical differences, p < 0.0001, were obtained for the tCho/tCr and tNAA/tCr ratios, reflecting a 2.5-fold increase in the tCho/tCr ratio and a two-fold decrease in the tNAA/tCr ratio in tumors. In line with these variations, the five-fold increase in the tumor tCho/tNAA ratio was also found to be statistically very significant. The 50% increase in the mI/tCr ratio in tumors was also found to be statistically significant with p < 0.05.

### CED of antitumoral drug and MRS acquisitions

**Figure 5:**
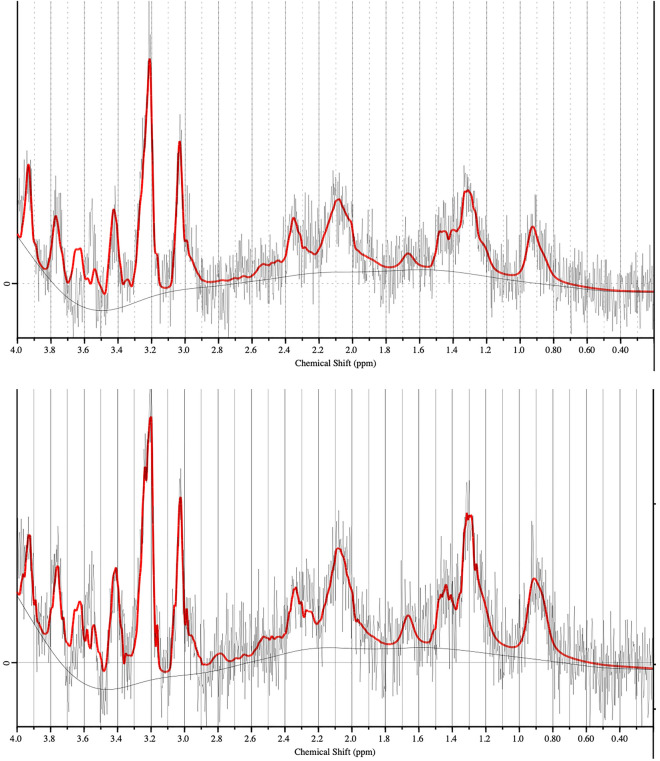
CED of choline kinase inhibitor and proton NMR spectra. Top and bottom proton spectrum were obtained in a C6 tumor of the same animal. Top spectrum was acquired before CED of RSM-9321A ChoK$\mathrm{\alpha }$ inhibitor solution. Bottom spectrum was acquired 1.5 h after starting the CED of RSM-9321A solution. Spectra were obtained using the microcoil as a receiver and a PRESS sequence with a total acquisition time of 8 min and 32 s. LC Model software was used for the identification and quantification of the metabolites. Grey and red curves correspond to the acquired non processed spectra and to the LCModel fitted spectra respectively.

After implantation of the NMR microcoil and capillary device, intratumoral CED of a 2 mM solution of RSM-932A ChoK$\alpha$ inhibitor^23,24^ was performed in six C6-bearing animals. As a control, an intratumoral CED of an aCSF solution was performed as well in two C6-bearing animals. The CED’s of the solutions were performed at a flow rate of 0.4 $\upmu$L/min. Proton MR spectra were acquired at baseline before the start of the CED procedure and were then acquired continuously during CED of the solution in the tumor. The spectra were acquired with the microcoil as a receiver using a PRESS sequence with TR/TE = 2000/15.266 ms and acquisition time of 8 min 32 s per spectrum. An example of proton spectrum evolution in C6 tumor during CED of ChoK$\alpha$ inhibitor and aCSF is shown in Fig. 5. The metabolite resonances were identified and quantified using the LC Model software. In this particular example, after 1.5 h of CED of RSM-932A solution, a 15% increase in the amplitude of lactate resonance at 1.3 ppm was observable, while a 7% decrease in the tCho resonance at 3.2 ppm and a 30% decrease of tNAA at 2 ppm were noticeable.

The spectra acquired during the CED of aCSF solution in the two sham animals showed no evolution in the amplitude of any resonance.

**Figure 6:**
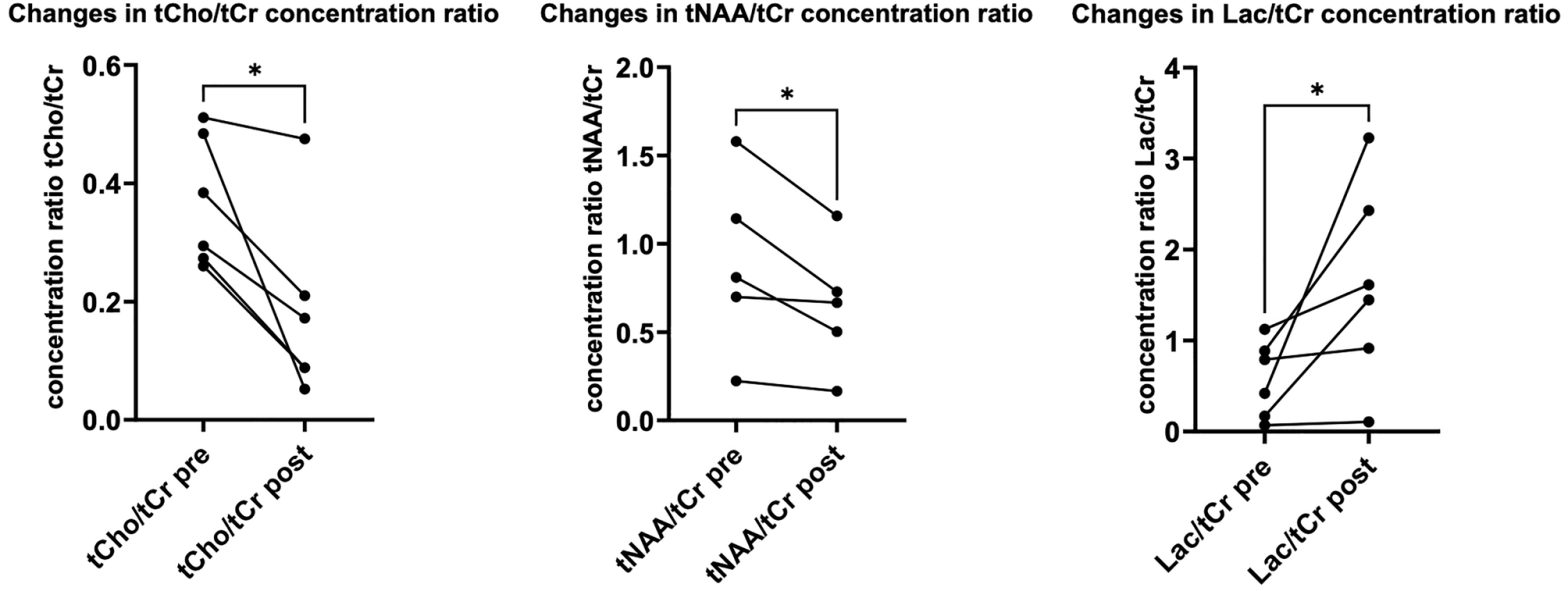
Changes in metabolite concentration under CED of choline kinase inhibitor. Evolution of the concentration, relative to the one of total creatine, of total choline, lactate and total NAA following the CED of RSM-9321A ChoK$\mathrm{\alpha }$ inhibitor. The ratios for these three metabolites demonstrated statistically significant changes following the CED with * corresponding to p < 0.05. N = 5 for tNAA/tCr concentration ratio due to the absence of detectable tNAA resonance in one of the animals.

In total, the evolution of eighteen metabolites was assessed using LC Model software following the infusion of ChoK$\alpha$ inhibitor. Three of them, tCho, tNAA and lactate/lipids, showed a significant change in their concentration relative to that of tCr. The evolution of the concentration of these three metabolites for the six C6-bearing animals with CED of ChoK$\alpha$ inhibitor is summarized in Fig. 6. The tCho/tCr ratio was observed to decrease by an average of 80% with a standard deviation (SD) of 53%. The tNAA/tCr ratio was also found to decrease by 31 ± 17% SD while the Lac/tCr increased by 83 ± 62% SD. The paired t-test applied to the ratio changes was found to be statistically significant with p-value of 0.026, 0.014 and 0.036 for tCho, tNAA and Lac/lipids, respectively.

## Discussion

Here we present an implantable device designed to allow simultaneous or delayed CED of drugs and localized high-resolution MRI and MRS at the administration site. The aim of the device is to help evaluate the local impact of drug administration on the metabolism using proton spectra and morphological and functional MRI. To this end, we chose to apply the device and evaluate its capabilities on an animal model of brain glioblastoma with the administration of choline kinase inhibitor and to investigate the impact in vivo of the drug on the tumor cell metabolism.

We have previously reported results obtained with brain-implanted microcoils of comparable size in healthy and tumor-bearing rats^14,15^. In these studies, the concentrations, relative to tCr, of tCho, glutamine, glutamate, lactate, mI, tNAA and taurine obtained in the brain of healthy animals with the implanted microcoil were similar to those reported in the literature using standard extracorporeal NMR coils. The results reported in these studies demonstrated the feasibility of the approach and the key role played by the miniaturization of the coil for enhancing the detection sensitivity on very small volume.

The NMR detection volume reported here for the implanted device was found to be similar to those measured previously^14,15^, of the order of 1 μL, when evaluated by FEM modeling or measured by MRI. Comparison of the detection sensitivity obtained with the microcoil of the implanted device and a commercial surface coil designed for rat brain confirmed the importance of miniaturization to achieve sufficient sensitivity in the vicinity of the capillary used for CED. The average FOG value of 119 obtained in vivo for five important proton resonance lines underlines the need to use an implanted microcoil to obtain usable proton MR spectra in a microliter volume within timescales compatible with in vivo conditions. The implanted microcoil of the device was also needed to acquire MRI images with in-plane spatial resolution of 26 μm, with acquisition times compatible with in vivo imaging. The MIP images illustrate the potential of implanted microcoil for probing the differences in vascularization between healthy cortex and the C6 glioma model where changes in microvasculature^18,19^ and more specifically an increase in vessel size index have been reported^25^.

As a first step in assessing the benefits of the implanted device, its NMR capabilities were tested on a group of control animals with healthy brain tissue and on a group of animals with gliomas. The differences observed in the NMR spectra between the two groups confirmed that the amplitudes of the choline and NAA resonances could be differentiated within the 1 μL detection volume of the implanted microcoil for acquisition times of the order of 8 min. Although quantitative comparisons are challenging due to differences in animal models and MRI protocols, these metabolic observations, namely an increase of tCho/tCr and mI/tCr ratios and a decrease of tNAA/tCr ratio in C6 tumor, are consistent with those previously reported in the literature on the C6 glioma model^26,27^.

In a second step, the implanted microcoil was used to visualize and validate the operability of the capillary for the CED of a solution, once positioned at the center of the microcoil. This validation was made possible by the high detection sensitivity of the microcoil, which enabled imaging of tissues located a few millimeters from the capillary. To highlight the distribution of the infused solution, its MRI visualization was enhanced using a Gd-based contrast agent. A high molecular weight (258 kDa) contrast agent was chosen to restrict its biodistribution to the interstitial compartment and to ensure as possible its tissue distribution via convective flow of the solution. Images obtained with the microcoil confirmed the effectiveness of CED, with homogeneous distribution of the highlighted solution in the volume of detection one hour after the start of CED.

In this study, the choice of molecule to evaluate the device as a whole was focused on the use of a choline kinase inhibitor and on its impact on the proton spectra. Choline is an important tumor biomarker metabolite whose large concentration in tumors is linked to cell proliferation in tumors and to the production of phosphatidylcholine (PC), an essential constituent of cell membranes. One of the enzymes in the PC synthesis pathway, choline kinase, enables the phosphorylation of free choline for synthetizing phosphocholine (PCho). Increased activity of ChoK$\alpha$, necessary for PC biosynthesis, has been observed in a variety of tumors, leading to an accumulation of PCho^28,29^. Based on these observations, CHK has naturally been identified as a promising therapeutic target for the treatment of cancer^30,31^. Among the ChoK$\alpha$ inhibitors, RSM-932A, also named TCD-717, has been evaluated on tumor-derived cell lines, on animal models of cancer and in patients with advanced solid tumors during a phase I clinical trial^23,24^. Following multiple intravenous administrations of RSM-932A and four weeks of treatment, Lacal et al.^24^ reported marked decrease (40%) in ChoK enzymatic activity and Pcho production in mice xenografts (human colon adenocarcinoma). Similarly, a sharp decrease of tCho/tCr concentration ratio after 90 min of CED of RSM-932A solution was observed in this study. The total amount of ChoK$\alpha$ inhibitor, delivered intratumorally to the animal after 90 min amounted to 0.5 mg/kg for the rats. For comparison, Lacal et al., performed RSM-932A intravenous injection at 3 mg/kg, three times per week during 4 weeks to the mice.

The rise in lactate/lipids NMR peak amplitude at 1.3 ppm that was observed in this study can be considered as another characteristic biomarker of the ChoK$\alpha$ inhibitor activity. These amplitude changes have previously been identified as markers of apoptotic induction in tumor cells and to the presence of different mechanisms of cellular response to the stress induced by the ChoK$\alpha$ inhibitor^32,33^.

CED of RSM-932A solution also resulted in a decrease in the tNAA/tCr amplitude ratio. The mechanisms of action behind this decrease in tNAA are, however, difficult to interpret due to the multiple functions in which NAA is involved.

The absence of noticeable spectra variations during CED of aCSF solution without ChoK$\alpha$ inhibitor ruled out the possible impact of the infused intratumoral volume of solution on the metabolic profile of the tumor.

The main objective of this study was to demonstrate the feasibility and value of using this implanted theranostic device for both delivery of therapeutic molecule and local assessment of its impact using high resolution MRI and metabolic MRS. This feasibility study focused on oncology and more specifically on brain glioma, but the scope of application of the device could be envisaged for other pathologies, in particular neurodegenerative diseases^34,35^, where the need for therapeutic solutions is considerable, and where MRI and MRS are the reference diagnostic techniques. The MRI images and MRS spectra acquired with the implanted microcoil could in principle benefit from many of the contrast mechanisms achievable with NMR, including diffusion, perfusion, magnetic susceptibility, blood oxygenation. In the preclinical field, the device could be advantageously used for screening and evaluation of drugs under development in animal models and could complement the standard use of MRI where tumor response is primarily assessed from changes in tumor size and do not reflect other functional, metabolic and non-morphological changes that may occur^36^. The device could also benefit patients by providing the possibility of adjusting and evaluating the efficacy of locally administered treatments.

While the use of the theranostic device opens the way to new applications, it also has potential limitations. Firstly, use of the device requires invasive procedures such as the placement of a cannula and the insertion of the device in the tissue. The accessibility of tumor lesions is another limitation to the applicability of the implanted device, although the feasibility of intratumoral chemotherapy or immunotherapy has been demonstrated in patients with a wide range of tumors, from brain, lung and pancreatic cancers^1–3,37,38^.

The third challenge, not addressed in this study, relates to the latency that may exist between the administration of therapeutic molecules and the observation of changes in images or NMR spectra. In the present study, changes in the metabolic profile occured rapidly after administration of the molecule and the effect of the molecule could be observed almost in real time with its administration. In a situation of delayed therapeutic response, the feasibility of longer-term implantation of the device should be considered and evaluated.

## Methods

### Implantable device fabrication

The NMR microcoils included in the implantable device were manufactured using insulated copper wires with 150 μm diameter (Tru Components 2UEWF, Conrad, Hirschau, Germany).

Constant (CMS 0805Weltron 460036 10 pF, Conrad, Hirschau, Germany) and variable amagnetic capacitors (Vishay BFC280905271 2-18 pF, RS COMPONENTS, Beauvais, France) were used for tuning and matching of the microcoil. A coaxial cable (RG 58 RP-SMA, Conrad, Hirschau, Germany) was used to connect the microcoil to the preamplifier of the NMR spectrometer. The capillaries used for drug CED were biocompatible polyimide capillaries (MicroLumen Medical Tubing, Oldslar, Florida, USA) with a 230 μm outer diameter and an inner diameter of 200 μm. Copper wire of the microcoil and capillary for CED were passed through a 1-cm long polyimide tubing (MicroLumen Medical Tubing, Oldslar, Florida) with an inner and outer diameter of 350 and 380 μm respectively. A biocompatible glue (Dentalon plus, Kulzer, Hanau, Germany) was used to seal this polyimide tubing.

### Animal studies

The study protocol was approved by the local animal welfare committee (University of Bordeaux, reference number 04490.02) and all animal experimental procedures were conducted in compliance with EU guidelines (directive 2010/63/EU) and the ARRIVE recommendations.

Animals were procured from Janvier Laboratory (Le Genest-Saint-Isle, France). They were kept in standard housing conditions (12 h light/dark cycles) with a standard rodent chow and water available ad libitum.

### In vivo experiments

Thirty rats, N = 15 male and N = 15 female, of Wistar strain (7 weeks of age, 160–180 g) were used for in vivo experiments. C6 gliomas were obtained by a stereotactic injection into the right barrel cortex (coordinates 0 mm AP, − 3 mm ML right hemisphere, − 2 mm DV)^39^ of C6 glioma cells (10^6^ cells) derived from *N*-nitrosomethylurea-induced rat glioblastoma (purchased from the ATCC-LGC Bank, Manassas, VA, USA).

Two 1-mm diameter holes were drilled in the skull for the placement of the cannula above the brain glioma and for the placement of a plastic screw. Screws and cannula were fixed to the skull with dental cement (Dentalon Plus, Kulzer, Hanau, Germany) on the surface of the skull. During surgery, rats were anesthetized through a facial mask (2.5% isoflurane in a mixture of air/O2 (70%/30%)) and positioned in a stereotaxic frame. After surgery, rats were housed individually and received doses of buprenorphine 0.05 mg/kg every 12 h for 48 h.

For the MRI and MRS experiment, the rats were first anesthetized in an animal chamber using a gas mixture of air and O_2_ with isoflurane (3%). During NMR acquisition, the animals were maintained under anesthesia with a gas mixture of air and O_2_ and isoflurane (1–2%). Body temperature was maintained around 37 °C by a hot water circuit positioned in the MRI cradle. During the acquisition, a breathing sensor was used to monitor the breathing rate of the animal and to adjust the adequate concentration of isoflurane for anesthesia. A syringe pump (CMA 402, CMA Microdialysis AB, Kista, Sweden) connected to the capillary inlet was used for intratumoral CED of the solutions.

RSM-932A (Sigma-Aldrich, St. Louis, MO, USA), 1,1′-[[1,1′-Biphenyl]-4,4′-diylbis(methylene)]bis[4-[(4-chlorophenyl)methylamino]-quinolinium dibromide (9CI), 1,1′-[[[1,1′-Biphenyl]-4,4′-diyl]bis(methylene)]bis[4-[(4-chlorophenyl)methylamino]-quinolinium] bromide, an inhibitor of choline-kinase $\alpha$ with molecular formula C_46_H_38_Cl_2_N_4_∙2Br and molecular weight of 877.53 Da was used in the study. The structural formula of the RSM-932A molecule is shown inFig. S4 as supplementary information.

The composition of the artificial cerebrospinal fluid (aCSF) solution used in the study was as follows: NaCl, 124 mM; KCl, 3 mM; CaCl2, 2 mM; MgSO4, 1 mM; KH2PO4, 1.25 mM; NaHCO3, 26 mM; pH 7.4.

The Gd-based contrast agent used for the visualization of CED of solution in vivo was a home-made synthetized polymer (258 kDA molecular weight) grafted with DOTAGA chelating Gd^+3^ ions^40^. The concentration of the polymeric contrast agent in the perfused solution was equal to 2 g/L and the corresponding concentration of Gd^3+^ ions in the solution was equal to 100 μM.

A timeline of the in vivo experiments for the healthy and tumor-bearing animal groups is shown in Fig. S5 as supplementary information.

### Radiofrequency simulations and characterization of microcoils

The distribution and the amplitude of the B_1_ RF magnetic field generated by the microcoil were simulated using the modeling software COMSOL Multiphysics 5.5 (COMSOL AB, Stockholm, Sweden) based on finite element method (FEM). The unloaded quality factor (the resonance frequency over the full width at half-maximum of the reflection coefficient) of microcoils was measured using a vector network analyzer (VNWA 3EC, SDR-Kits, Melksham, United Kingdom). The quality factor of the loaded coils positioned within the magnet was measured using the network analyzer of the MRI spectrometer.

### MRI and MRS protocols

Magnetic resonance measurements were performed on a preclinical 7-T Bruker BioSpec 70/20 MRI (Bruker BioSpin, Ettlingen, Germany) running under Paravision 6.0.1. Quadrature birdcage coil with inner diameter of 86 mm (Bruker, Ettlingen, Germany) was used as RF transmitter. Receive-only 2 × 2 rat brain surface array coil (Bruker, Ettlingen, Germany) was used as a reference coil to evaluate the performance of the microcoils.

Magnetic resonance spectra were acquired using a single-voxel PRESS (point-resolved spectroscopy) sequence with the following parameters: echo time TE = 15.3 ms; repetition time TR = 2000 ms; voxel size = 2 × 4 × 2 mm^3^. The VAPOR (variable pulse powers and optimized relaxation delays) module was used for water peak suppression.

A ZTE (zero echo time) sequence was used to acquire fast and isotropic 3D volumes with 2.5 ms TR, 9 × 9 × 9 mm^3^ FOV (Field of View) and total acquisition time of 2 min. A 2D multislice FLASH (Fast Low Angle SHot) sequence was used for in vivo imaging with TR/TE = 200/3.5 ms and 10 × 10 mm^2^ FOV.

The microcoils were centered and positioned vertically within the volume coil. The normal to the elliptical surface of the microcoil was placed perpendicular to the B_0_ static magnetic field. No active decoupling of the microcoil was used during RF excitation. For shimming, a 3D magnetic field distribution was first acquired with the transmit volume coil using the MAPSHIM protocol. Localized first and second order automatic shims were then performed using the microcoil as a receiver.

### MR spectrum processing

The SNR values of the metabolites in solution were obtained using the TopSpin software (Bruker, Ettlingen, Germany).

MR spectra obtained in control animals and C6-bearing animals for comparison of metabolite amplitudes were processed using a java-based version of the MR user interface package jMRUI. A first-order phasing and a Lorentzian apodization of 5 Hz were performed and the residual water components were removed using the HLSVD (Hankel-Lanczos Singular Value Decomposition) algorithm. The Quest (QUantum ESTimation) module was used for the identification of the resonance of the metabolites. Spectra obtained in animals were fitted and quantified with the QUEST (quantitation based on Quantum ESTimation) procedure from jMRUI^41^. A basis set of 12 metabolite spectra was simulated at 7T for the in vivo experimental protocol using the NMR Scope-B. The basis set included choline and phosphocholine (tCho), lactate (Lac), creatine and phosphocreatine (tCr), *N*-acetylaspartate and *N*-acetylaspartylglutamate (tNAA), glutamate (Glu), glutamine (Gln), myo-inositol (mI) and taurine (Tau). Only peak quantifications with a Cramer-Rao lower bound greater than 20% of the SNR were considered sufficiently reliable^42^.

LCModel (Linear Combination of Model spectra) software^43^ was used to estimate metabolite peak areas during CED of ChoK$\alpha$ solution. To compute the absolute quantification of each metabolite, the experimental spectrum was fitted between 0.5 and 4.1 ppm with a group of basis sets for the spectrum of a specific metabolite for the PRESS proton MRS sequence for a TE equal to 15 ms. No baseline correction and zero filling were applied to the in vivo data prior to analysis in LCModel.

### Statistical analysis

The software environment R for statistical computing and graphics was used for the comparison of metabolite concentration ratio between control animals and C6 tumor-bearing animals.

Analysis of spectra evolution during CED of solutions in C6-glioma was performed using Prism software (GraphPad Software, Boston, MA, USA).

### Supplementary Information

Supplementary Figures.

## Data Availability

The datasets used and/or analyzed during the present study are available from the corresponding author upon reasonable request.
