# Supplementary material for: Implantable theranostic device for in vivo real-time NMR evaluation of drug impact in brain tumors
Source: Sci Rep. 2024 Feb 24;14:4541. doi: 10.1038/s41598-024-55269-1 (PMC10894190; doi:10.1038/s41598-024-55269-1)
Supplement: Supplementary file 1 — Supplementary Figures. [file 41598_2024_55269_MOESM1_ESM.docx]

**Supplementary information**


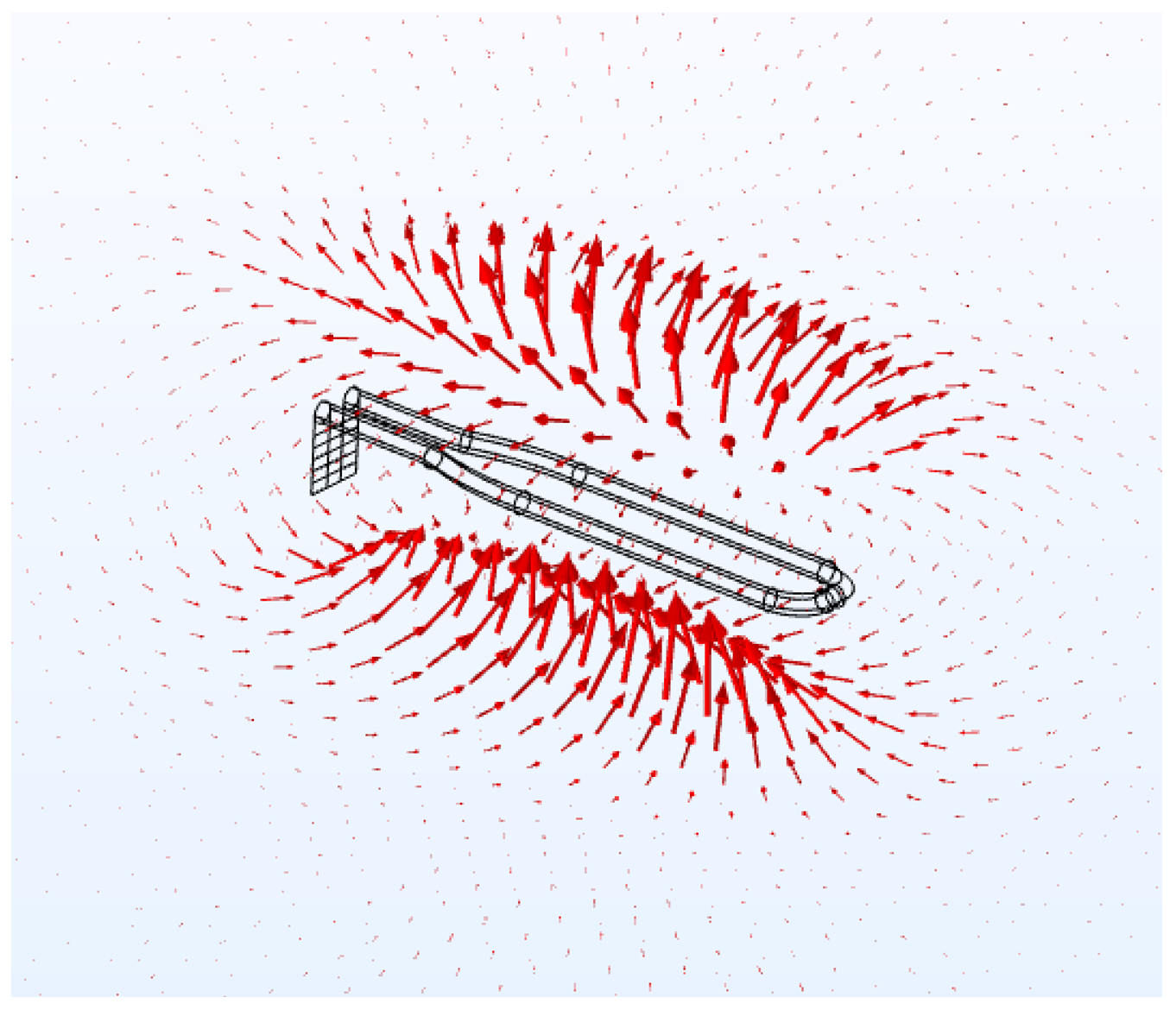

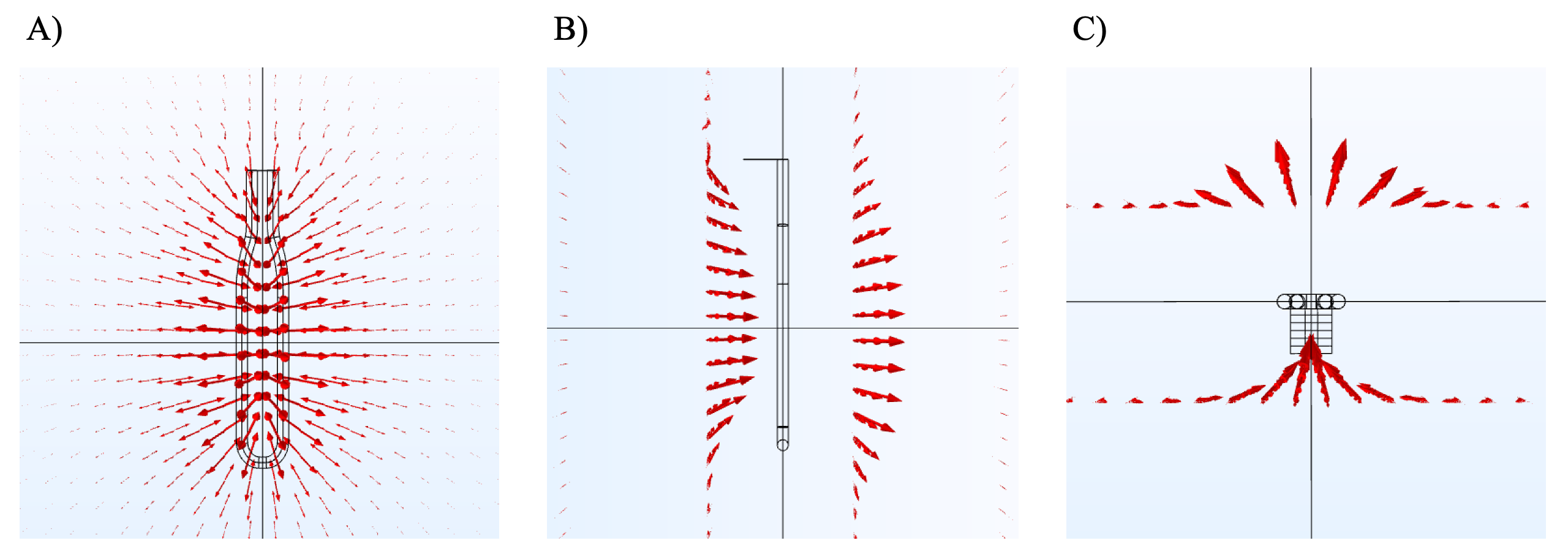


Fig. S1. 3D display of the B1 magnetic field generated by the microcoil obtained with a FEM modeling software. Red arrows are proportional to the B1 amplitude. (oblique view on the left, view from above the ellipse-shape coil on the right)


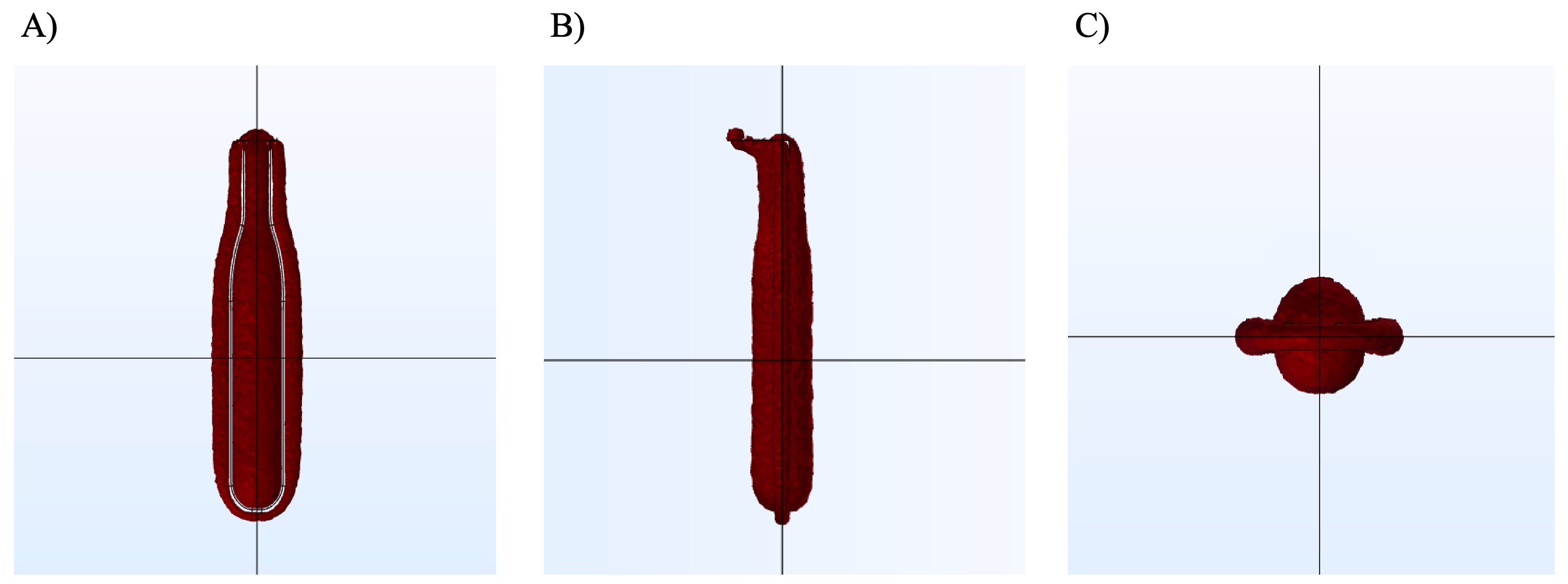


Fig. S2 Isosurface of the amplitude of the transverse component, relative to the static magnetic field, of the B_1_ RF field seen in three orthogonal planes, (A) ellipse-shape view, (B) side view and (C) top view. Regions without B1 values in (A) correspond to the coil wire.


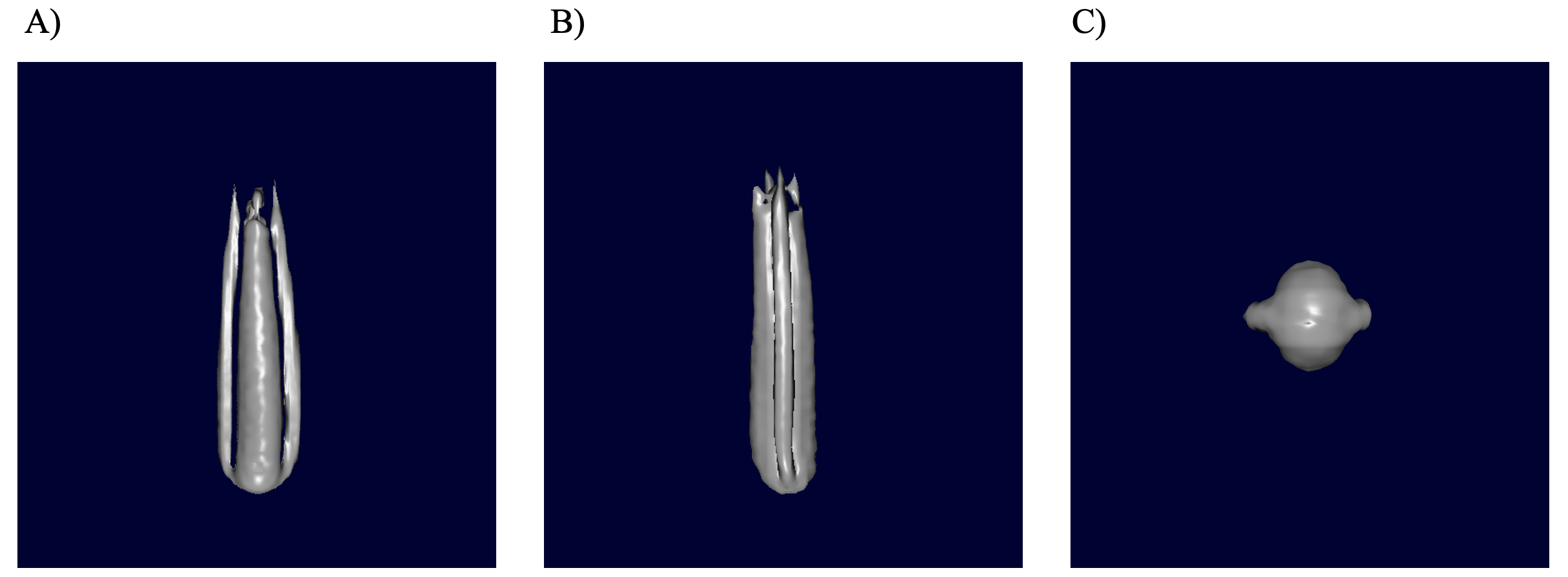


Fig. S3. Volume rendering of a 3D MRI acquisition obtained with the microcoil. The three views correspond to the orientation of the isosurface shown in Fig. S2.


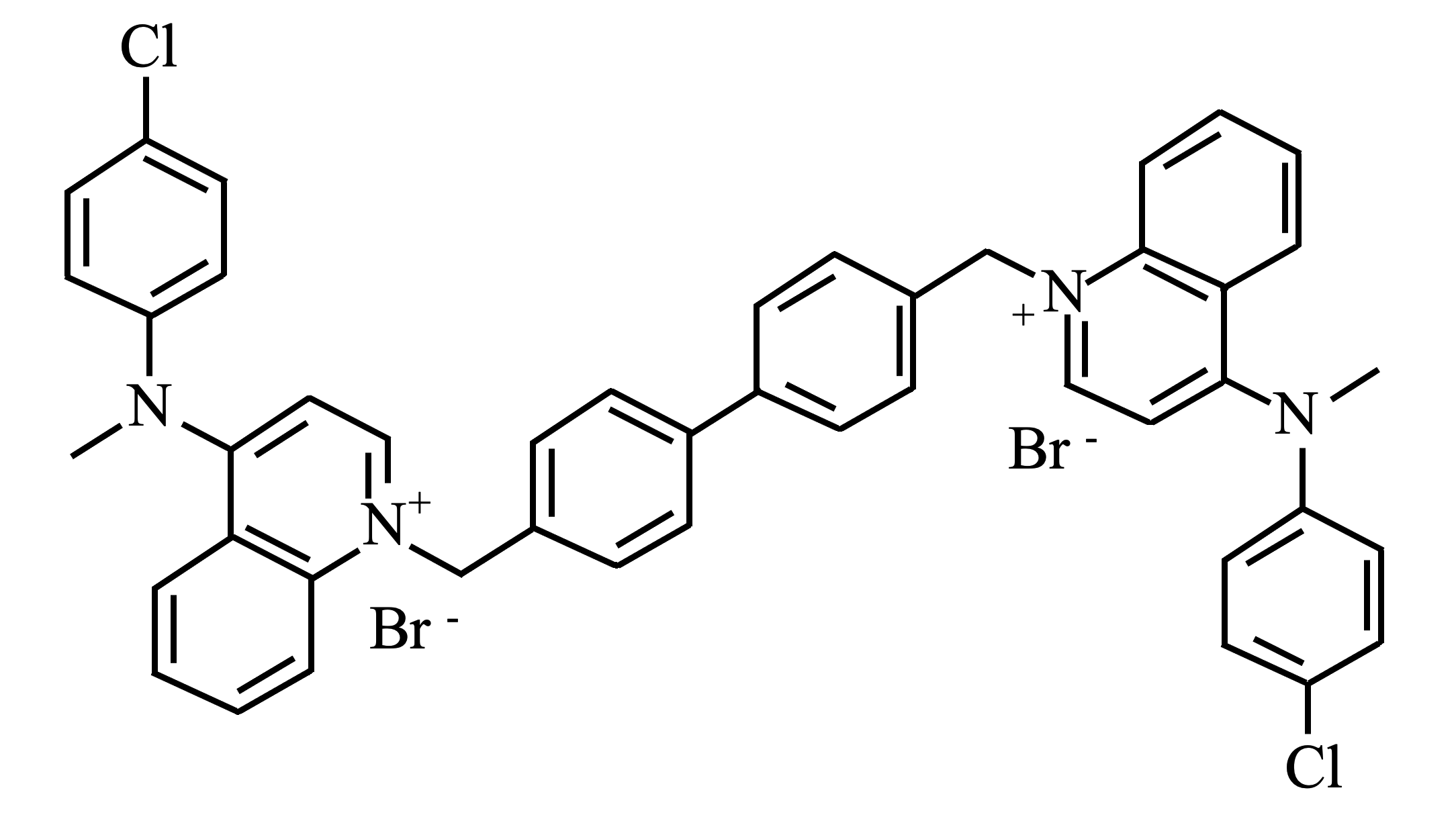


Fig. S4. Structural formula of the RSM-932A ChoK α inhibitor.


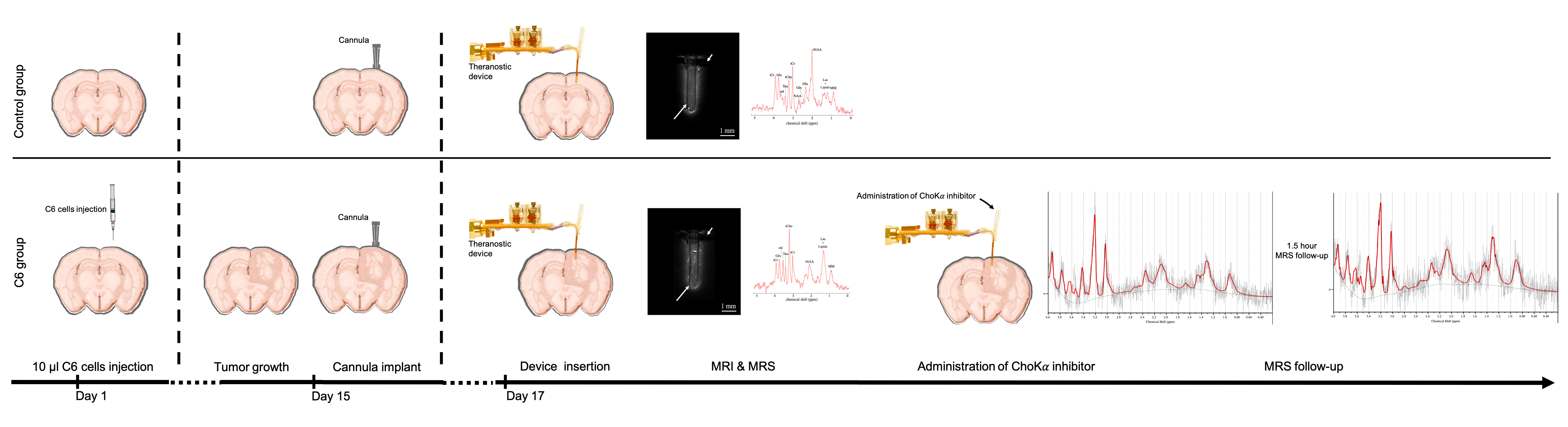


Fig. S5. Timeline of the in vivo experiments for the control and C6 tumor-bearing groups..
